# *Foxf* Genes Integrate *Tbx5* and Hedgehog Pathways in the Second Heart Field for Cardiac Septation

**DOI:** 10.1371/journal.pgen.1004604

**Published:** 2014-10-30

**Authors:** Andrew D. Hoffmann, Xinan Holly Yang, Ozanna Burnicka-Turek, Joshua D. Bosman, Xiaomeng Ren, Jeffrey D. Steimle, Steven A. Vokes, Andrew P. McMahon, Vladimir V. Kalinichenko, Ivan P. Moskowitz

**Affiliations:** 1 Departments of Pediatrics, Pathology, and Human Genetics, The University of Chicago, Chicago, Illinois, United States of America; 2 Department of Pediatrics, Cincinnati Children's Hospital Medical Center, Cincinnati, Ohio, United States of America; 3 Department of Molecular Biosciences, Institute for Cellular and Molecular Biology, University of Texas, Austin, Texas, United States of America; 4 Department of Regenerative Medicine and Stem Cell Biology, University of Southern California, Los Angeles, California, United States of America; Stanford University School of Medicine, United States of America

## Abstract

The Second Heart Field (SHF) has been implicated in several forms of congenital heart disease (CHD), including atrioventricular septal defects (AVSDs). Identifying the SHF gene regulatory networks required for atrioventricular septation is therefore an essential goal for understanding the molecular basis of AVSDs. We defined a SHF Hedgehog-dependent gene regulatory network using whole genome transcriptional profiling and GLI-chromatin interaction studies. The Forkhead box transcription factors *Foxf1a* and *Foxf2* were identified as SHF Hedgehog targets. Compound haploinsufficiency for *Foxf1a* and *Foxf2* caused atrioventricular septal defects, demonstrating the biological relevance of this regulatory network. We identified a *Foxf1a* cis-regulatory element that bound the Hedgehog transcriptional regulators GLI1 and GLI3 and the T-box transcription factor TBX5 *in vivo*. GLI1 and TBX5 synergistically activated transcription from this cis-regulatory element *in vitro*. This enhancer drove reproducible expression *in vivo* in the posterior SHF, the only region where *Gli1* and *Tbx5* expression overlaps. Our findings implicate *Foxf* genes in atrioventricular septation, describe the molecular underpinnings of the genetic interaction between Hedgehog signaling and *Tbx5*, and establish a molecular model for the selection of the SHF gene regulatory network for cardiac septation.

## Author Summary

Atrioventricular septal defects (AVSDs) are a common severe class of congenital heart defects. Recent work demonstrates that events in the second heart field (SHF) progenitors, rather than in the heart, drive atrioventricular (AV) septation. Our laboratory has shown that both Hedgehog signaling and the T-box transcription factor, Tbx5, are required in the SHF for AV septation. To understand the molecular underpinnings of the AV septation process we investigated SHF Hedgehog-dependent gene regulatory networks. Transcriptional profiling and chromatin interaction assays identified the Forkhead box transcription factors *Foxf1a* and *Foxf2* as SHF Hedgehog targets. Compound haploinsufficiency for *Foxf1a* and *Foxf2* caused AVSDs in mice, demonstrating the biological relevance of this pathway. We identified a cis-regulatory element at *Foxf1a* that bound TBX5 and Hedgehog transcriptional regulators GLI1 and GLI3 *in-vivo*. Furthermore, TBX5 and Gli1 co-activate transcription of the identified cis-regulatory element *in-vitro*. The enhancer is expressed primarily in the pSHF in-vivo, where *Tbx5* and *Gli1* expression overlap. Our findings implicate Foxf1a and Foxf2 in AV septation and establish Tbx5 and Hedgehog signaling upstream of *Foxf* genes in a gene regulatory network for cardiac septation.

## Introduction

Cardiac septation, the morphogenetic process that transitions the looped heart tube into the multi-chambered heart observed in mammals, is complex and often goes awry in Congenital Heart Disease (CHD). Atrioventricular septation is the crucial process that separates the common atrioventricular canal into right and left compartments. Atrioventricular septal defects (AVSDs) are a common severe form of CHD. A novel paradigm for the developmental ontogeny of the atrioventricular septum has recently emerged [1]–[6]. This work describes atrioventricular septation as a process driven by molecular events in second heart field (SHF) cardiac progenitors rather than in the heart itself [1]–[6]. The identification of extracardiac lineages that generate the atrial and atrioventricular septum implies that the search for gene regulatory networks germane to cardiac septation should occur in SHF cardiac progenitors not in the heart itself.

Hedgehog signaling is an essential developmental pathway conserved from flies to man [7], [8]. Mutations in key Hedgehog pathway genes, including ligands such as *Sonic hedgehog* (*Shh*; 20423) and downstream signaling cascade member *Smoothened* (*Smo*; 319757) cause significant cardiac defects including complete atrioventricular septal defects [9], [10]. Tissue specific knockout of Hedgehog signaling in the SHF recapitulates atrioventricular septal defects [4], [5] and genetic inducible fate mapping showed that the atrial/atrioventricular septum is derived from Hedgehog-receiving SHF cardiac progenitors [5]. These observations laid the groundwork for identifying the Hedgehog-dependent SHF gene regulatory networks essential for atrial septation.

Cardiogenic transcription factor genes *Tbx5* (21388), *Nkx2.5* (18091) and *GATA4* (14463) have been implicated in human atrial septation [11]–[14]. These transcription factors form a complex and can co-activate gene expression [12], [15]–[17]. *Tbx5* has been shown to be required in multiple contexts during cardiac development and adult function in mice. *Tbx5* is required in the SHF for atrioventricular septation [6], [15], in embryonic cardiomyocytes for proliferation [18], in adult myocardium for contractile function [19], and in the adult cardiac conduction system for cardiac rhythm control [20]. Tbx5 target genes differ significantly between these distinct cellular and temporal contexts [6], [21]. Yet the Tbx5-responsive cis-regulatory elements specific to these cellular contexts and the molecular cues that establish context dependent selectivity remain unknown.

We previously described genetic interactions between *Tbx5* and Hedgehog signaling in the SHF for atrioventricular septation in mice [6]. Mice haploinsufficient for both *Tbx5* and the obligate Hedgehog signaling receptor gene *Smo* express AVSDs more frequently than mice haploinsufficient for either gene alone [6]. Furthermore, constitutive Hedgehog signaling in *Tbx5*-mutant SHF progenitors can rescue atrioventricular septation [6]. These studies predict that Hedgehog-dependent and Tbx5-dependent gene regulatory networks share vital, yet undescribed overlap in the SHF that is necessary for atrioventricular septation.

In this study we attempted to define Hedgehog-dependent SHF gene regulatory networks and identify the molecular basis of the genetic interaction between Hedgehog signaling and Tbx5. We characterized the Hedgehog-dependent SHF gene regulatory networks by *in vivo* whole genome transcriptional profiling and GLI-chromatin interaction studies. We found that *Foxf1a* (15227) and *Foxf2* (14238) are downstream of Hedgehog signaling in the SHF. Mice haploinsufficient for both *Foxf1a* and *Foxf2* compound heterozygotes have atrial septal defects, demonstrating the biological relevance of these Hedgehog targets. GLI3T (14634) binding data identified a candidate cis-regulatory element upstream of *Foxf1a* that contained an adjacent Tbx5 binding site. This enhancer binds to GLI1 (14632), GLI3 and TBX5 in the SHF *in vivo*. *In vitro* and *in vivo* analysis demonstrated that this cis-regulatory element integrates Hedgehog signaling with *Tbx5* activity and provides strong specific activity in the posterior SHF. This work identifies a novel role for Foxf transcription factors at the intersection of *Tbx5* and hedgehog signaling in atrioventricular septation and describes a SHF gene regulatory network for cardiac morphogenesis.

## Results

### Transcriptional profiling of the posterior SHF in *Shh* mutants

**Figure 1:**
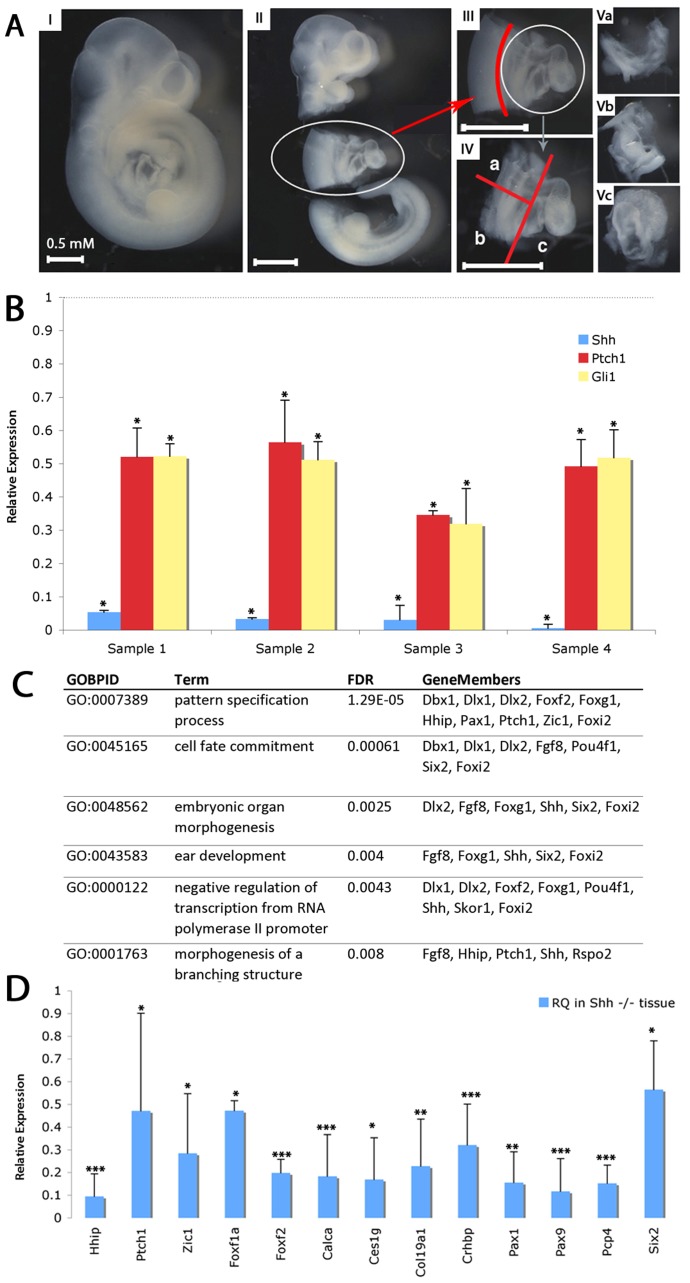
Transcriptional profiling of SHF from *shh^−/−^* embryos. (A) Microdissection for isolation of SHF tissues. E9.5 embryos were isolated (I). Thoracic tissues including the heart were removed from head and tail, kept for genotyping or non-cardiac controls (II). Neural tube was removed (III). SHF tissue was bisected and separated from the heart (IV). Microdissected tissue was kept as anterior SHF (Va), posterior SHF (Vb) or heart (Vc). (B) RT-PCR demonstrates decreased expression of *Shh*, *Gli1* and *Ptch1* in *shh* mutant SHF tissues isolated for transcriptional profiling (C) Gene Ontology biological processes (GOBPs) enriched in the transcriptional profile analysis of SHF tissue from wild-type and *Shh* mutant embryos identifies developmental terms. (D) 13 genes identified in the transcriptional profile were verified as Shh-dependent using RT-qPCR (relative quantitation, RQ). * indicates p<0.05, ** indicates p<0.01, *** indicates p<0.001.

Progenitor cells for the atrial and atrioventricular septum require Shh signaling in the posterior SHF (pSHF) between embryonic day 8 and embryonic day 10 (E8–E10) to migrate into the heart to form the atrial septum between E9–E11 [4], [5]. To identify the Hedgehog-dependent gene regulatory networks required for this process, we compared transcriptional profiling of the posterior SHF from wild-type and *Shh* (MGI: 1932461) null embryos at E9.5 to identify differentially expressed transcripts. We isolated the pSHF by microdissection including the dorsal mesenchymal protrusion and closely associated surrounding ventral lateral plate mesenchyme. Our dissection included the attached foregut, but excluded the heart, dorsal lateral plate mesenchyme and neural tube (Figure 1A). RNA was isolated and known Hedgehog-dependent transcripts were evaluated by RT-PCR to verify genotyping prior to whole genome transcriptional profiling. *Shh*, *Ptch1* (19206) and *Gli1* all demonstrated significantly reduced expression (p>0.05) in the *Shh* null samples compared to wild-type micro-dissected samples (Figure 1B). Specifically, *Shh* was reduced more than 90%, while *Ptch1* and *Gli1* were each reduced approximately 50%, consistent with significantly reduced *Hedgehog* signaling in the mutant samples and confirming the genotypic fidelity of the isolated samples.

Transcriptional profiling of pSHF samples was performed on Agilent Mouse Whole Genome Arrays. Using a significance threshold with a multi-test adjusted p-value (Q-value) <0.005 and absolute fold change larger than 2, comparing *Shh^−/−^* mutant mouse embryos (n = 4) with wild-type embryos (n = 3) identified a differentially expressed 560-gene signature (Table S1). Gene Ontology (GO) enrichment analysis of differentially expressed genes captured known processes disrupted in Hedgehog pathway mutants, such as pattern specification and organ morphogenesis (Figure 1C) [22]. To further identify the best candidates for an experimental validation, 65 genes were computationally evaluated according to more stringent criteria by three statistical tests (non-parameter Wilcox-tested theoretical p<0.15, empirical t-tested FDR<0.1, and absolute fold change>3, Figure S1) on the same data sets. From the Shh down-regulated candidates, we chose 21 targets and validated significant misexpression of 13 by qPCR (p<2e-16, Fisher's Exact test, FET) (Figure 1D). Eight others did not meet criteria for statistically significant misexpression primarily due to large expression variation, possibly related to the presence of non-SHF tissue isolated by our dissection process.

### Identification of Hedgehog signaling direct targets in the SHF

**Figure 2:**
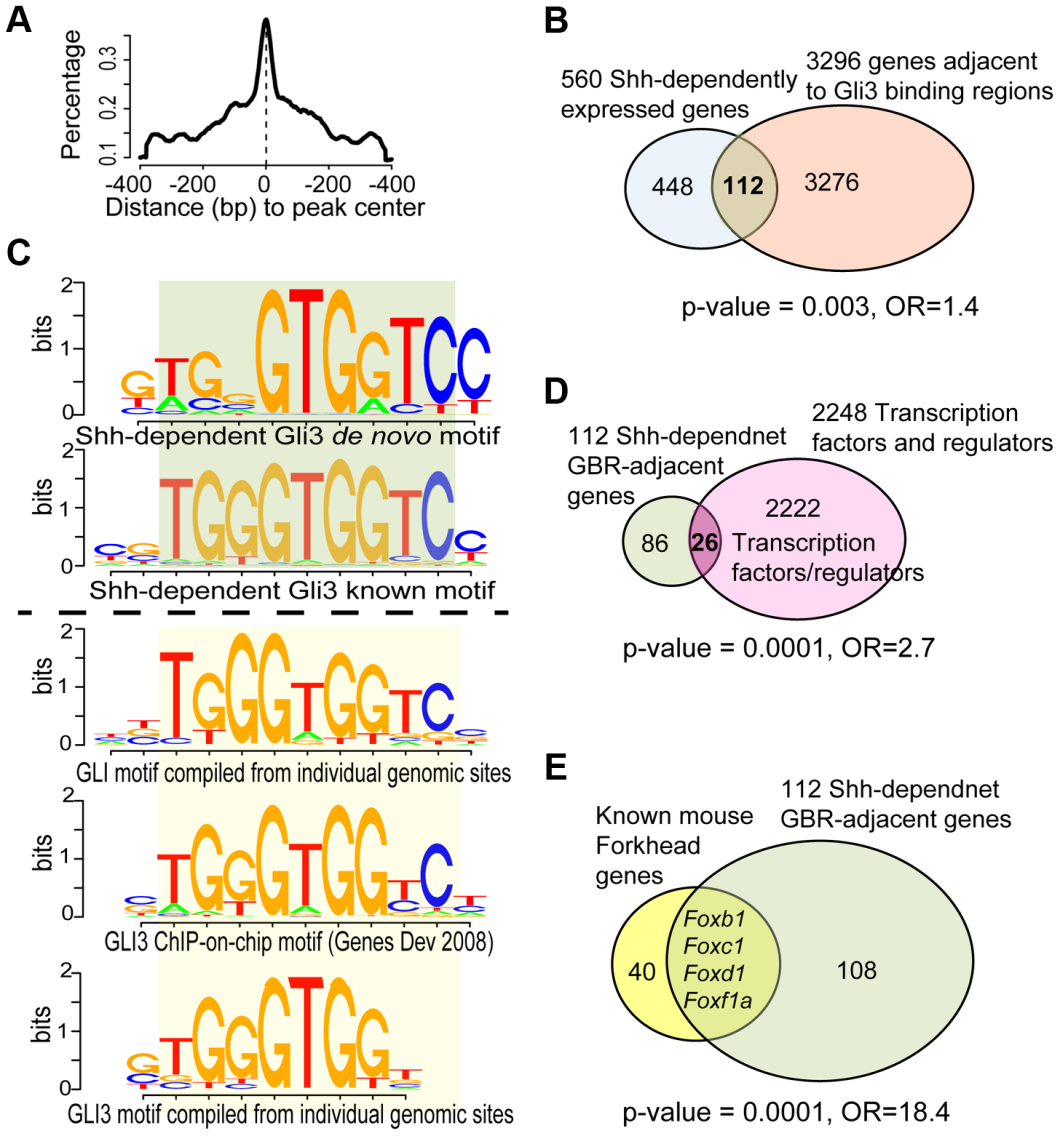
Analysis of ChIP-Seq data and its intersection with transcriptional profiling data. (A) Distribution of ChIP-seq peaks highlighted the modeled GLI3-binding centered in peak regions, using MACS2 software. (B) GLI3 ChIP-seq revealed 1316 peaks defining potential binding sites in the mouse genome. Intersection with *shh*-dependent transcriptional profiling identified 112 candidate direct Hedgehog-dependent target genes. (C) Summary of *de novo* and known motifs enriched in *shh*-dependent GLI3-bound regions (Top 2 sub-panels) compared with similar known GLI motifs from literature and TRANSFAC database (Bottom 3 sub-panels). (D) Among the 112 genes, 26 are transcription factors or regulators of transcription, a significant over-representation. (E) Among the 112 genes, 4 are FOX family transcription factors, a significant over-representation.

To define loci directly downstream of Hedgehog signaling, we analyzed genome-wide chromosomal binding locations of the Hedgehog transcriptional regulator Gli3 in the embryonic SHF by chromatin immunoprecipitation with deep sequencing (ChIP-seq). We performed ChIP using a Cre-inducible flag-tagged Gli3T expression line (RosaGli3T^Flag c/c^ MGI: 3828280) [23] combined with the SHF Cre driver *Mef2c-AHF-Cre* [24] (MGI: 3639735). The SHF tissue from 50 *Mef2cAHF*-*Cre* ^+^; *Rosa^Gli3TFlag/+^* embryos was micro-dissected and immunoprecipitated using an anti-FlagM2 antibody (Sigma). To verify enrichment of Gli3T bound sequences by immunoprecipitation prior to sequencing, we tested a previously identified Gli3T peak upstream of *Ptch1* (Chromosome 13, nucleotides 63577408–63579384, mm9), a known Gli3T-bound cis-regulatory element in the limb [23]. This sequence was 13.7-fold enriched in the SHF IP fraction by ChIP-PCR. We proceeded to sequence the IP library and apply Model-based Analysis for ChIP-Seq (MACS) [25]. We identified 1316 Gli3-bound peaks by comparing 68 million sequence tags in IP to 21 million sequence tags in input (tag size = 36 bps, effective genome size = 2e+9, band width = 200, 2<model fold<200, and p-value cutoff = 1e-05) [25]. From these peaks, we analyzed the distribution of the signal around the peak center and identified a typical distribution, confirming successful sequencing (Figure 2A). The predominant GLI3T peak location from the binding data was intergenic and a considerable distance from the transcriptional start sites. We therefore considered the possibility that genes and up to 100 kbp in both directions from intergenic peaks may fall under control of GLI-mediated cis-regulatory elements, given that enhancers often reside thousands of base pairs away from their target of regulation and act independently of their orientation [26], [27]. We therefore annotated GLI3T-bound regions to all transcription start sites within 100 kbp and to the nearest TSS if it resided outside the 100 kbp window [28], [29]. This consideration resulted in mapping the 1316 peaks to 3296 neighbor genes (Table S2). The enrichment between GLI3T-bound and Shh-dependent genes was significant among approximately 22,000 mouse genes (FET p<0.01, Figure 2B).

To define the direct Hedgehog-dependent SHF gene regulatory networks, we intersected the SHF Shh-dependent transcriptional profile signature with the SHF Gli3T chromatin contact results to define candidate Hedgehog-dependent Gli-target genes. This dataset intersection comprised 119 peaks annotated to 112 genes (Figure 2B, Table S3). The enrichment between Gli3T-bound and Shh-dependent genes was significant among ∼22k mouse genes (FET p = 0.003, odds ration = 1.4, Figure 2B). The 119 Shh-dependent Gli3T-bound sites contained significant enrichment of the *de novo* and known Gli3-binding motif, as derived by ChIP-Chip (CGTGGGTGGTCC) [23] and by computational implication (TRANSFAC database; Figure 2C, bottom panel) [30], [31] at a high degree of significance (p≤1e-10; Figures 2C, top panel).

A significant enrichment of transcription factors was observed in SHF Hedgehog target genes. Transcription factor activity and DNA binding were the two most significant gene-sets over-represented among the 112 Shh-dependent Gli3-bound genes. We directly analyzed our gene set for overrepresentation of transcription factors by searching TRANSFAC version 2013.1 [31] and identified 26 TFs among the 112 unique genes with significant Gli3T-bound peaks (Figure 2D, Table S4), representing a significant enrichment (p = 0.0001, odds ratio = 2.7, Fishers exact test). Specifically, Shh transcriptional profiling and GLI3T chromatin interaction data both identified an enrichment of FOX gene family members, encoding Forkhead transcription factors, identifying FOX genes as potential SHF Hedgehog targets (Figure S2). The set of 112 Shh-dependent Gli3T bound genes included four Fox transcription factors, *Foxb1* (64290), *Foxc1* (17300), *Foxd1* (15229) and *Foxf1a*, representing a significant enrichment (Figure 2E, p = 0.0001, odds ratio = 18.4).

### *Foxf1a* and *Foxf2* are downstream of *Shh* in the SHF

**Figure 3:**
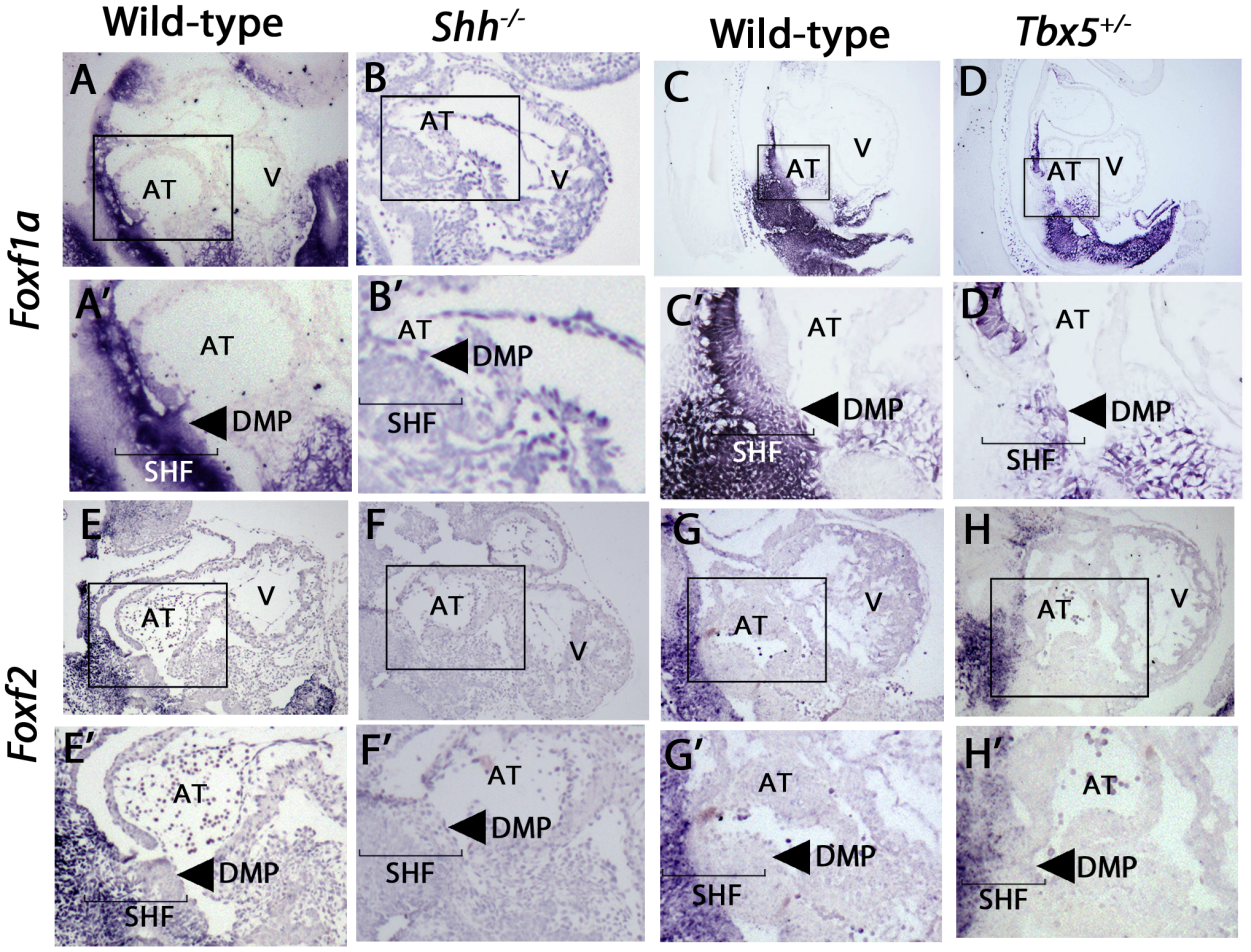
Expression of *Foxf1a* and *Foxf2* in *shh^−/−^* and *Tbx5^+/−^* mutants embryos at E9.5. In-situ hybridization demonstrated SHF expression of both *Foxf1a* and *Foxf2*, with a loss of SHF expression of *Foxf1a* in *Shh* mutants, (A, B) and a near-complete loss of *Foxf2* in *Shh* mutants (E, F). *Tbx5* heterozygotes expressed *Foxf1a* at decreased levels specifically in the posterior SHF tissues (C, D), whereas *Foxf2* expression patterns were unchanged (G, H). Arrow: dorsal mesenchymal protrusion tissues in A′–H′, Brackets: SHF mesenchymal tissues. AT: Atrium, V: Ventricles.

We investigated the hypothesis that *Foxf1a* and *Foxf2* expression was downstream of Hedgehog signaling in cardiac development. *Shh*-dependent expression of both genes in the SHF was confirmed by qPCR: *Foxf1a* expression was reduced by 50% (p = 0.05) and *Foxf2* was reduced by 80% in the SHF of *Shh^−/−^* versus wild-type controls (p = 0.01) (Figure 1D). *In-situ* hybridization to evaluate the patterning of expression showed that *Foxf1a* and *Foxf2* were both expressed in the posterior SHF, but not in the heart, in wild-type embryos at E9.5, with *Foxf1a* expression extending more ventrally than *Foxf2* to include the DMP (Figures 3A, A′, E, E′). Mesenchymal expression of both *Foxf1a* and *Foxf2* demonstrated a severe decrement in *shh^−/−^* mutant embryos (Figures 3B, B′, F, F′).

In a search for common targets between *Tbx5* and Hedgehog signaling in the SHF, we tested whether *Foxf1a* and/or *Foxf2* SHF expression was *Tbx5-*dependent. We performed *in situ* hybridization for *Foxf1a* and *Foxf2* in *Tbx5* ^+/−^ heterozygous mutant embryos (MGI: 2387850), which demonstrate 40% penetrance of AVSDs [15]. *Foxf1a* but not *Foxf2* expression demonstrated significant reduction in *Tbx5* heterozygotes at E9.5. In *Tbx5* ^+/−^ embryos, *Foxf1a* expression was specifically decreased in the posterior SHF (Figure 3C, C′, D, D′, arrow) in the area of expression overlap with *Tbx5* expression [6]. In regions where *Foxf1a* expression does not overlap with *Tbx5* expression, such as the anterior SHF, *Foxf1a* expression appeared normal (Figure 3D, D′). *Foxf2* expression in *Tbx5* ^+/−^ embryos appeared unaltered compared to wild-type embryos (Figure 3G, G′, H, H′). Taken together, this analysis demonstrates that posterior SHF *Foxf1a* expression was *Shh*- and *Tbx5*-dependent whereas *Foxf2* pSHF expression was *Shh*-dependent alone.

### *Foxf1a* and *Foxf2* are required for atrioventricular septation

**Figure 4:**
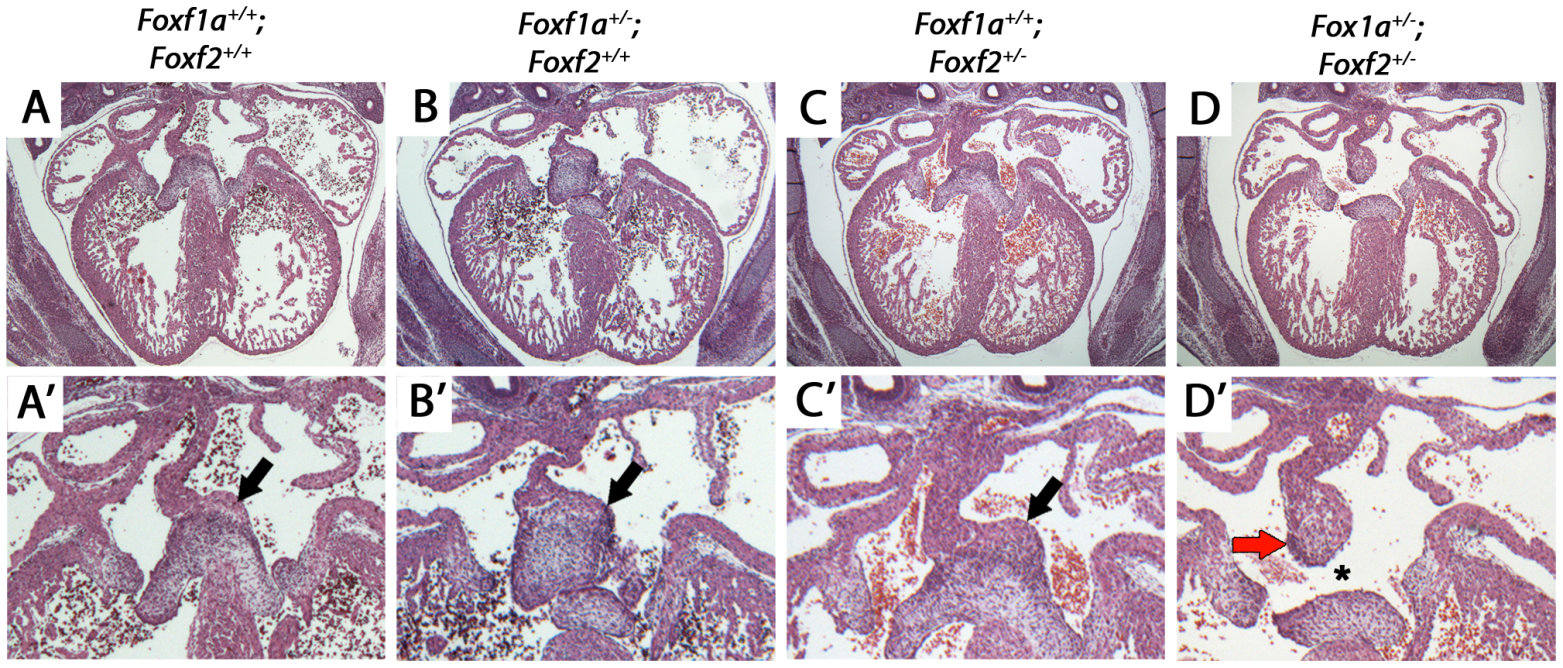
Atrioventricular septal defects in *Foxf1a^+/−^; Foxf2^+/−^* compound heterozygote embryos at E14.5. *Foxf1a^+/−^; Foxf2^+/−^* embryos displayed atrial septal defects including absence of the dorsal mesenchymal protrusion (D, D′, black arrows). Compound heterozygotes also displayed expanded mesenchymal cap of primary atrial septum (red arrow) (D, D′). Wild-type (A, A′), *Foxf1a^+/−^* (B, B′), and *Foxf2^+/−^* embryos (C, C′) showed no atrial septal defects. P-values (Fisher's exact test): *Foxf1a* ^+/−^ (9 embryos) vs wild-type (4 embryos) = 1; *Foxf2* ^+/−^ (2 embryos) vs wild-type = 0.33; *Foxf1a^+/−^; Foxf2^+/−^* (3 embryos) vs wild-type = 0.03.

We hypothesized that *Foxf1a* and *Foxf2* were required in a dosage sensitive manner for atrioventricular septation. We analyzed the cardiac anatomy of embryos from an intercross between *Foxf1a* ^+/−^ and *Foxf2* ^+/−^ at E14.5, when cardiac septation is normally complete. *Foxf1a^+/−^*; *Foxf2* ^+/−^ double-heterozygote embryos all exhibited atrioventricular septal defects (Figure 4D, D′ asterisk; p = 0.03). Primum-type atrial septal defects, characterized by absence of the dorsal mesenchymal protrusion, were observed in each case (Figure 4D, D′). Additionally, *Foxf1a* ^+/−^; *Foxf2* ^+/−^ double-heterozygotes displayed larger than normal mesenchymal caps covering the primary atrial septum (Figure 4D′ arrow), an observation in keeping with the known redundant requirement for *Foxf1a* and *Foxf2* in limiting mesenchymal growth in other contexts [32]. Atrial septal defects were never observed in *Foxf1a* ^+/−^ (Figure 4B, B′) or *Foxf2* ^+/−^ (Figure 4C, C′) single-heterozygotes or wildtype control littermate embryos (Figure 4A, A′). We concluded that *Foxf1a* and *Foxf2* are redundantly required for atrioventricular septation.

### A cis-regulatory element at *Foxf1a* binds TBX5, GLI1, and GLI3 *in vivo*

**Figure 5:**
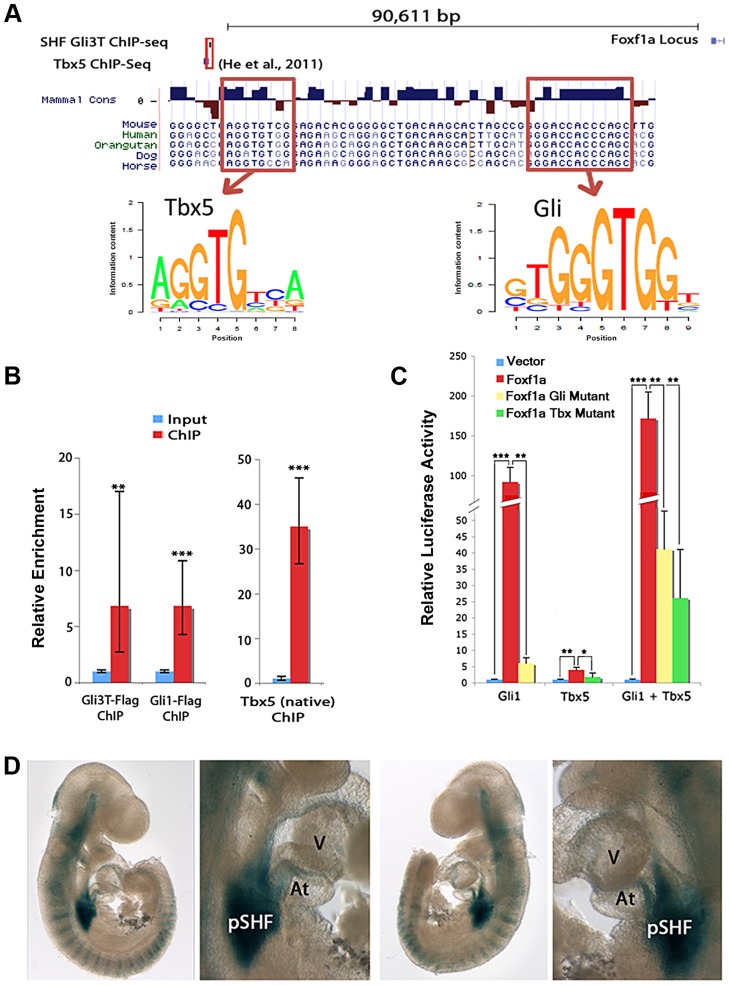
Integration of Hedgehog and Tbx5 activity on an enhancer at *Foxf1a*. (A) Integration of Hedgehog and Tbx5 activity on an enhancer at *Foxf1a*. ChIP-seq for GLI3 (Figure 2) and TBX5 [35] identified a candidate *Foxf1a* enhancer. (B) ChIP-PCR from microdissected pSHF for GLI3, GLI1 and TBX5 demonstrated *in vivo* binding of each factor to the candidate enhancer. (C) Luciferase assays demonstrated that GLI1 and TBX5 individually and together synergistically activated the enhancer. Activation of enhancer with mutated GLI binding sites was significantly reduced by GLI1; however, synergistic GLI1/TBX5 activity is largely maintained. Activation of enhancer with mutated TBX binding sites was reduced cells transfected with TBX5 alone, but activation in cells transfected with both GLI1 and TBX5 was still relatively high. (D) Representative images of the enhancer activated specific posterior SHF expression of *lacZ* in transient transgenic embryos at E9.5. Atria: At; Ventricle: V. P-values:, * indicates p<0.05, ** indicates p<0.01, *** indicates p<0.001.

We hypothesized that *Foxf1a* may represent a direct downstream target of Hedgehog signaling and/or Tbx5 in the SHF. We identified *Foxf1a* as a candidate direct target based on unbiased interrogation of GLI3T and TBX5 transcription factor chromatin interaction and transcriptional profiling data sets. We intersected our SHF GLI3T ChIP data set (Figure 2B) with a published TBX5 ChIP-seq data set generated from HL-1 cardiomyocytes [33] to define regions with potential co-occupancy of both transcription factors. The intersection of the ChIP-seq datasets identified a single overlapping interaction peak for Gli3T (in the SHF (Figure 2B)) and TBX5 (in HL-1 cardiomyocytes) [33] located approximately 90 kb upstream of the *Foxf1a* transcription start site (Figure 5A and Figure S3). The *Foxf1a* transcriptional start site is the closest protein-coding gene to the described peak. The transcriptional start site for a non-coding RNA is located approximately 1.3 kbp upstream of *Foxf1a*, oriented in the opposite direction [34]. Closer interrogation of the sequence underlying the interaction domains revealed a conserved canonical T-box binding site (AGGTGTGG; chr 8, nucleotides 123,517,714–721, NCBI137/mm9 assembly) and a conserved canonical Gli binding site (GGACCACCCAGC; chr 8, nucleotides 123,517,754–762, NCBI137/mm9 assembly) within 30 base pairs of one another (Figure 5A). We evaluated the sequence information content for these sites from our SHF Gli3 ChIP-seq experiment and found close agreement with published binding sites for Gli3 [23], [33]. This chromatin interaction data in combination with the *Tbx5* and Hedgehog signaling-dependent *Foxf1a* SHF expression (Figure 3) identified this conserved region (mouse chromosome 8, nucleotides 123,517,714–762) as a candidate *Foxf1a* cis-regulatory element.

We evaluated the binding of TBX5 and the Hedgehog transcriptional regulators GLI1 and GLI3 to the candidate cis-regulatory element at *Foxf1a in vivo* in the SHF. We evaluated TBX5 binding *in vivo* by performing ChIP using an anti-TBX5 antibody on the micro-dissected wildtype SHF at E10.5 and observed 35-fold enrichment of the cis-regulatory element in the TBX5-immunoprecipitation fraction compared to the input fraction by qPCR (Figure 5B). We evaluated GLI1 and GLI3T binding *in vivo* by performing ChIP on the micro-dissected SHF of mice carrying either a *Cre*-activated flag-tagged *Gli3* (*RosaGli3T^Flag c/c^*) or *Gli1* allele (*RosaGli1^Flag c/c^* MGI: 4460761) in concert with the *Nkx2.5-Cre* (MGI 2654594), broadly expressed cardiac tissues and progenitors. We performed ChIP using an anti-flag antibody on the SHF from R26R-Gli3-flag*^Nkx2.5-Cre/+^* or R26R-Gli1-flag*^Nkx2.5-Cre/+^* embryos at E10.5 and observed, respectively, 6.8-fold and 7.1-fold enrichment of the *Foxf1a* cis-regulatory element in the GLI1- and GLI3T-overexpressing embryos over the input control by qPCR (Figure 5B). We also evaluated two genomic loci between our identified binding site and the *Foxf1a* transcription start site to determine whether nonspecific pulldown occurred in our ChIP experiments. These loci were not significantly enriched in the IP'd DNA (Figure S4) These results validate *in vivo* SHF binding of TBX5, GLI1, and GLI3 to the candidate cis-regulatory element at *Foxf1a*.

### A cis-regulatory element at *Foxf1a* integrates Tbx5 and Hedgehog activity

We hypothesized that the conserved, adjacent, and functional *in vivo* Gli and Tbx5 binding sites may integrate *Tbx5* and Hedgehog activity as a component of a downstream gene regulatory network. We evaluated the activity of TBX5 and GLI1 on the candidate *Foxf1a* enhancer *in vitro*. The conserved element was cloned into a pGL4.23 vector containing a minimal promoter driving luciferase as a transcriptional readout and was transfected into HEK293T cells along with expression vectors for Gli1 and/or Tbx5. Co-transfection with the expression vector for Gli1, a Hedgehog-responsive transcriptional activator, provided a 91.9-fold induction of luciferase activity (p = 0.0017). Co-transfection with the expression vector for Tbx5 alone provided a 3.9-fold increase of luciferase activity (p = 0.039). Co-transfection with both Gli1 and Tbx5 expression constructs provided a 171.6-fold increase in luciferase activity (p = 0.00091), demonstrating synergistic activity between these transcriptional co-activators (Figure 5C).

We assessed the requirement of TBX5 and GLI binding sites for transcriptional activation of the enhancer. To assess the requirement of TBX5-dependant transcriptional activation of the enhancer on TBX5 binding sites, a TBX5-mutant enhancer-luciferase construct with the 7 base pair core of 3 canonical TBX binding sites was generated by site-directed mutagenesis. This TBX5-mutant construct eliminated transcriptional activation by TBX5 alone (p = 0.04) and limited transcriptional activation by TBX5 and GLI1 together (p = 0.006) (Figure 5C). A GLI-mutant enhancer-luciferase construct was also constructed with the 8 base pair core of 3 canonical binding sites altered by site-directed mutagenesis (see materials and methods). This GLI-mutant construct profoundly diminished transcriptional activation by GLI1 alone (p = 0.001) (Figure 5C). Interestingly, transcriptional activation by TBX5 and GLI1 on the GLI-mutant enhancer construct was only modestly abrogated luciferase compared to the activity of GLI1 and TBX5 on the wild-type enhancer (p = 0.003).

### SHF-specific *in vivo* expression of the cis-regulatory element at *Foxf1a*

We hypothesized that the cis-regulatory element at *Foxf1a* may integrate Hedgehog signaling and Tbx5 activity as a SHF-specific enhancer *in vivo*. We cloned the *Foxf1a* genomic region into an *Hsp68*-*LacZ* expression construct, whose minimal promoter affords no intrinsic *in vivo* expression activity [35]. We evaluated the enhancer activity of the *Foxf1a* genomic fragment by evaluating *LacZ* expression in transient transgenic mouse embryos at E9.5. The posterior SHF demonstrated strong *lacZ* expression and was the only anatomic region demonstrating consistent and robust expression in the 8 transgenic embryos genetically positive for *LacZ* (Figure 5D). Interestingly, the SHF region with the most consistent and robust expression was the area of overlap between Hedgehog signaling and Tbx5 expression [6], including the early dorsal mesenchymal protrusion and surrounding mesenchyme of the posterior SHF (less frequent and intense expression was also observed in other anatomic locations that receive Hedgehog signaling outside of the Tbx5 expression domain, including the anterior SHF (5/8), anterior lateral plate mesoderm (5/8) and somites (2/8) (Figure 5D). These observations, in concert with the *in vitro* analysis suggested that Hedgehog and Tbx5 act synergistically to provide strong reproducible transcriptional activation of this enhancer in the posterior SHF.

## Discussion

**Figure 6:**
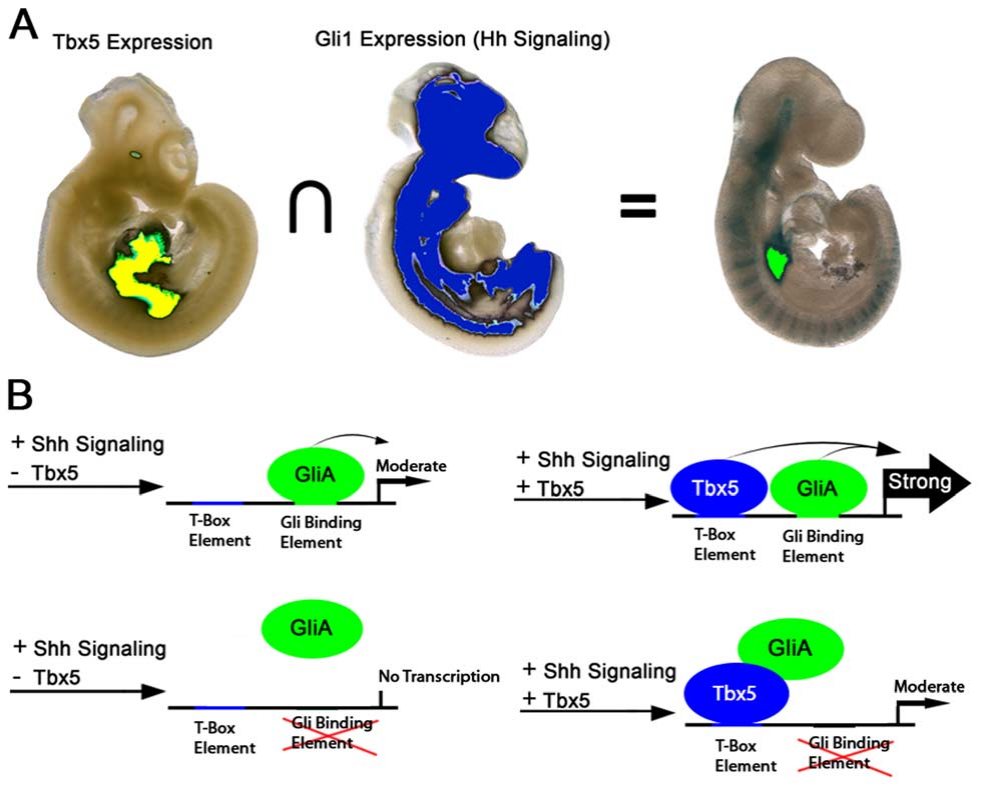
Model for Hedgehog/Tbx5 interaction. (A) Intersection of *Tbx5* expression, restricted to the posterior SHF and heart, and *Gli1* expression, broadly expressed in axial mesenchyme and brain but excluded from the heart, is the posterior SHF. Activation of TBX5/GLI1 responsive enhancer is observed principally in the overlap between the *Tbx5* and *Gli1* expression domains. (B) In the presence of GLI activator (GLIA) alone, the enhancer is weakly active. In the presence of both GLIA and TBX5 is transcription from the enhancer strongly activated. When the GLI binding site is mutated, GLIA alone is insufficient to activate strong expression, but GlLIA may interact with TBX5 to activate expression more strongly than TBX5 alone.

Identification of the gene regulatory networks required for atrioventricular septation will be the basis for a mechanistic understanding of AVSDs, a common severe form of CHD. We investigated Hedgehog-dependent networks and implicated *Foxf* genes for the first time in vertebrate heart development. We examined the overlap between Hedgehog pathways and Tbx5, both known to be integral in the pSHF for atrioventricular septation. Using transcription factor-chromatin interaction data, we identified a cis-regulatory element at *Foxf1a* that bound both TBX5 and the Hedgehog pathway transcriptional activator GLI1. *In vitro* analysis of TBX5 and GLI activity on the cis-regulatory element at *Foxf1a* proved predictive of *in vivo* biology: this enhancer exhibited strong transcriptional activation only in the pSHF region where Tbx5 expression and Hedgehog signaling intersect. This region is the location of atrial septum progenitors [5], where both Hedgehog signaling and Tbx5 are required for atrioventricular septation (Figure 6) [4]–[6]. These observations provide molecular detail for the genetic interaction between Tbx5 and Hedgehog signaling in atrioventricular septation.

### Foxf transcription factors are required in the SHF for atrial septation

Our observations identified a requirement for the Forkhead-box transcription factors *Foxf1a* and *Foxf2* in heart development. Compound haploinsufficiency for both *Foxf1a* and *Foxf2* caused an atrial septal defect of the primum type, an atrioventricular septal defect characterized by absence of the dorsal mesenchymal protrusion. *Foxf1a* and *Foxf2* were expressed selectively in the SHF, not in the heart (Figure 3). The requirement for *Foxf* genes in atrioventricular septation (Figure 4) provided further support for a model of atrioventricular septation as driven by molecular events in SHF cardiac progenitors as opposed to in the heart itself. We found that *Foxf1a* and *Foxf2* are required downstream of Hedgehog signaling in atrioventricular septation, adding cardiac development to the previously described Hedgehog-dependent role for *Foxf* genes in murine gut development [32], [36]–[37]. Atrioventricular septal defects are also observed in *Shh*-null mutant embryos [10]. Because *Foxf1a* and *Foxf2* expression were each decreased in the SHF by more than 50% in *shh^−/−^* null embryos (Figure 1D), *Foxf1a* ^+/−^; *Foxf2* ^+/−^ double heterozygote embryos provided a reasonable developmental facsimile of their diminished expression levels in *shh^−/−^* embryos. The observation that *Foxf1a* and *Foxf2* compound haploinsufficiency resulted in AVSDs is therefore consistent with the supposition that *Foxf1a* and *Foxf2* are essential components of the Hedgehog-dependent SHF gene regulatory network.

*Foxf* genes have also been previously implicated in cardiac specification in the ascidian *Ciona intestinalis* [38], [39]. In ascidians, the single *Foxf* orthologue lies at the center of a pathway regulating numerous migration-related cellular processes, such as polarity, migration and membrane protrusion in trunk ventral cardiac progenitor cells [38], [39]. *Ciona* trunk ventral cells with disrupted Foxf activity fail to migrate properly, but still differentiate into cardiac tissue at an improper location. Interestingly, removing Hedgehog signaling from the mouse SHF causes a migration failure of SHF progenitors [4], [5]. Like the *Ciona* trunk cells without *Foxf*, SHF cells without Hedgehog responsiveness differentiate into cardiomyocytes, but their altered migration causes AVSDs [5]. Future efforts will determine whether the requirement for *Foxf* genes in cardiac progenitor migration is a conserved feature of mammalian cardiac development.

### A SHF cis-regulatory element integrates Tbx5 and Hedgehog pathways: Building a gene regulatory network for cardiac septation

Genetic interaction and rescue experiments investigating the requirement for Hedgehog signaling and Tbx5 in atrioventricular septation were consistent with Tbx5 acting either in parallel or upstream of Hedgehog signaling in atrioventricular septation [6]. Our interrogation of these pathways on a cis-regulatory element at *Foxf1a* provides molecular detail for their interaction. We observed that TBX5 and GLI1, the Hedgehog-dependent transcriptional activator, synergistically activated the cis-regulatory element *in vitro* (Figure 5C) predicting strong activation of expression in areas of overlap between Tbx5 expression and Hedgehog signaling. This prediction held *in vivo*, where transcriptional activity of the enhancer was strong and reproducible only in the posterior SHF region, where Tbx5 expression and Hedgehog signaling overlap (Figures 5D and 6). This work is consistent and a model describing a SHF-specific gene regulatory network driven by GLI1 and TBX5 and essential for atrioventricular septation (Figure 6). This model provides specific predictions for the logic underlying enhancer choice in the SHF with ramifications for understanding the molecular and biochemical basis of atrioventricular septation and clinical AVSDs.

## Materials and Methods

### Ethics statement

Mouse experiments were completed according to a protocol reviewed and approved by the Institutional Animal Care and Use Committee of the University of Chicago, in compliance with the USA Public Health Service Policy on Humane Care and Use of Laboratory Animals.

### Mouse lines and handling

The *Shh^−^* line was obtained from the Jackson laboratory. The Tbx5^+/−^ mice have been previously reported [15]. *Foxf1^+/−^* and *Foxf2^+/−^* mouse lines were generated in the Kalinichenko lab (Cincinnati Children's Hospital Medical Center) by breeding *Foxf1a^fl/fl^* and *Foxf2^fl/fl^* mice with EIIA-Cre transgenic mice (Jackson Lab). Mef2c-AHF-Cre [24], ROSA26-Gli1 [40] and ROSA26-Gli3T [23] were reported previously.

### Dissection techniques

For ChIP, transcriptional profiles and in-situ hybridizations, embryos were dissected in nuclease-free PBS on ice. For SHF microdissection procedures, head tissues anterior to the heart were removed, as were tail tissues posterior to the heart. Portions of these tissues were retained for genotyping if necessary. Neural tube tissues were also removed. The SHF mesenchyme was bisected into anterior and posterior portions when necessary, and then removed from the cardiac tissue (Figure 1A).

### Transcriptional profiling

*Shh^+/+^* and *Shh^−/−^* embryos were dissected as described above at E9.5. SHF tissues from these embryos were pooled to isolate sufficient amount of RNA for synthesis of labeled cRNA. Transcriptional profiles were performed using Agilent Mouse Whole Genome Arrays *mgug4122a*.

### ChIP

Microdissected SHF tissues were grouped into pools of approximately 50. Tissues were briefly fixed in 1.8% formaldehyde, then washed and homogenized. Sonication was performed with a Misonix 4000 sonicator until the sheared chromatin was approximately 100–300 bp in length. Input control samples were reserved prior to overnight immunoprecipitation with the appropriate antibody bound to magnetic Dynabeads (Invitrogen). Beads were precipitated and washed, the chromatin was eluted, de-crosslinked and purified using a PCR cleanup kit (Qiagen). To determine fold enrichment, qPCR was performed using input controls compared with DNA bound to immunoprecipitated proteins, using primers specific to the site of interest as well as primers to two sites not expected to be enriched.

### Gli3-bind peaks and the enriched motifs derived from ChIP-seq

#### RNA extraction and ChIP-seq

To prepare the ChIP-seq library, chromatin was fixed and sonicated to 300–500 bp fragments, then was immunoprecipitated, eluted, de-crosslinked and column-purified before submission for sequencing. High-throughput sequencing was performed on Illumina Genome Analyzer following the manufacturer's protocols. The raw data was deposited in GEO with an accession number GSE44755.

#### Binding peaks

Gli3T ChIP-Seq immunoprecipitated product (IP) was compared to the input from SHF tissue dissected from mouse embryos at E9.5. The raw IP sequence reads were first trimmed 8 bps on the left end for two reasons: 1) they showed unexpected low quality and 2) they were 8 bps longer than the input reads. Then we used Bowtie aligner software to map ChIP-seq and control sequencing reads to the mouse reference genome build mm9. The genome-wide locations of Gli3-binding peaks were identified using a model-based analysis of ChIP-seq (MACS) algorithm version 2.

#### Motif identification

Motifs were identified using HOMER software by the default parameters. Visualization was conducted using R and a local mirror of the UCSC Genome Browser with customized data.

#### Candidate Gli3-targets annotation

The Gli3-bound sites were first annotated to the mouse mm9 assembly genome by HOMER software to the nearest transcription start sites (TSSs). Additional genes were included following analyses that localized them to the same chromosome within 100 kbp distance to any Gli3T-bound peaks. These genes were annotated using Bioconductor packages *biomaRt* version 2.14.0 and *ChIPpeakAnno* version 2.6.0.

### Shh-dependent transcriptomic alteration derived from microarray

#### Data pre-process

Expression of the pooled RNAs (8 Shh^−/−^ pools and 7 Shh^+/+^ pools) were extracted by Feature Extraction Software (v. 10.5.1.1) available from Agilent, using the default variables. Outlier features on the arrays were flagged by the Bioconductor [41] package *Agi4x44PreProcess* and were excluded. Raw feature intensities (the meanSignals) were background corrected, varance stabilizing normalization (VSN) normalized and log2 transformed [42]. As has been noted in previous studies, non-expressed probes are merely background noise and thus no longer track the expression of genes, lower expressed probes were filtered to increase true positive on the array [43]. As a result, 11,469 probes (encoding 8,867 out of 20,674 genes on the array) were retained for signature identification that met the following three criteria: 1) Met a minimum criterion of signal quality flagged by Agilent Feature Extraction; 2) Cross-sample expression fell into the top half of the inter-quartile range (IQR); 3) Presented known genes as annotated by Biocondctor package *mgug4122a.db* version 2.6.3. The raw data was deposited in GEO with an accession number GSE44754.

#### Data analysis

To identify Shh-dependent gene signature, the whole mouse genome gene-expression was compared between *Shh* mutant samples and wide-type controls, using R and Bioconductor package *samr* (unpaired two-class t-statistic, 100 permutations) [44]. The resultant p-values of all genes with at least 2-fold changes (computed by *samr*) were corrected for multiple testing using the Q-value adjustment. Differentially expressed genes were identified that had a Q-value less than 0.5%. The hypergeometric test for all Gene Ontology biological processes was performed using Bioconductor package *GOStats*.

### Tbx5-dependent microarray transcriptomic data analysis

#### Data pre-process

Analysis performed was identical to that used to identify Shh-dependent transcriptomic alterations. Briefly, VSN [42] was used to do between arrays normalization, and the resultant expression measurements were log2 transformed. The raw data was deposited in GEO with an accession number GSE43599. A Q-value <0.05 indicated significantly differentially expression.

#### Candidate Tbx5 and Gli3 co-targets

Tbx5-bound peaks derived from ChIP-seq were recently published using HL-1 cells [33]. These Tbx5 binding sites were annotated by HOMER software to the nearest transcription start sites (TSSs) in the mouse mm9 reference genome. As T-box motif is significantly enriched among the 119 Shh-dependent and Gli3-binding peaks in SHF, we further searched for candidate Tbx5 and Gli3 co-targets, using HOMER software (Figure 5A).

We checked the intersection of transcriptomic altered genes dependent on either Tbx5 or Shh that intersected with Tbx5 and Gli3 bound genes. The list of resultant genes includes those with S*hh*- or *Tbx5*-dependent expression on the microarray experiments and being located within 100 kbp distance to a Gli3-bound region derived from the ChIP-seq experiment. These genes were further annotated using Bioconductor package biomaRt version 2.14.0.

### Data accession

ChIP-seq and microarray data were deposited in the Gene Expression Omnibus (GEO) database with a super accession number GSE44756.

### *In-situ* hybridization

*In-situ* hybridization was performed as in Moorman et al. [45] with slight modifications. Specifically, after post-hybridization washes with 50% formamide/2X SSC, specimens were incubated for 30 minutes at 37 degrees C in 20 ug/ml RNase A to remove unbound probe and reduce nonspecific staining. All *in-situ* hybridization experiments were performed on a minimum of three control and three experimental embryos.

### Luciferase assays

Expression vectors for *Gli1* and *Gli3T* were obtained from the Vokes lab. *Tbx5* was cloned into the pCDNA 3.1 expression construct [20] *Foxf1a* fragment was cloned into the pGL4.23 vector (Promega). Expression and reporter vectors were transfected into HEK293T cells using FuGENE (Promega). Cells were cultured for 48 hours after transfection, then lysed and assayed using the Dual-Luciferase Reporter Assay system (Promega).

### Transient transgenics

The *Foxf1a* enhancer and minimal promoter used in the luciferase assays were subcloned from the pENTR vector into the Hsp68-LacZ vector [35] using the Gateway system (Invitrogen). The resulting construct was digested with NotI enzyme to remove the pBlueScript backbone, gel-purified, injected into fertilized mouse eggs at the University of Chicago Transgenics Core Facility and implanted into female mice. Embryos were harvested at E9.5 and stained as described previously [5].

## Supporting Information

Figure S1 Optimized *shh*-dependent candidates for in vitro validation. Panel A) 65 genes generated from all three statistical tests on the same data are interrogated. Panel B) Hierarchical classification of samples based on the expression of these 65 genes splits *Shh* mutants from wild types.
(TIF)

Figure S2 Known mouse Forkhead-box genes are enriched among the identified *shh*-dependent and Gli3T-bound genes.
(TIF)

Figure S3 Browser views of the actual sequence mapping profiles between the identified *Gli3*-bound site and *Foxf1a* TSS. The two top panels show the estimated enriched peaks and the density measurements respectively. The two bottom panels are the genome (mm9) coordinates and the RefGenes.
(TIF)

Figure S4 Genomic regions near the *Foxf1a* gene but without identified Gli or Tbx binding sites were tested in Gli1-Flag, Gli3-Flag, and Tbx5 ChIP samples as controls for specificity. Neither of these sites significantly amplified over input controls, suggesting that the Gli and Tbx5 ChIP was specific for the identified enhancer.
(TIF)

Table S1 A 560-gene signature (640 probesets) was identified by comparing shh−/− mutant mouse embryos (n = 4) with wild type embryos (n = 3).
(XLS)

Table S2 The identified 1316 significant Gli3-bound regions.
(XLS)

Table S3 The 112 Shh-dependent and SHF Gli3-bound genes (119 peaks).
(XLS)

Table S4 The 26 are transcription factors according to TRANSFAC database version 2013.1, among the 112 Shh-dependent Gli-bound genes.
(XLS)
