# Correction: *Foxf* Genes Integrate *Tbx5* and Hedgehog Pathways in the Second Heart Field for Cardiac Septation

**DOI:** 10.1371/journal.pgen.1006533

**Published:** 2016-12-29

**Authors:** Andrew D. Hoffmann, Xinan Holly Yang, Ozanna Burnicka-Turek, Joshua D. Bosman, Xiaomeng Ren, Linglin Xie, Jeffrey D. Steimle, Steven A. Vokes, Andrew P. McMahon, Vladimir V. Kalinichenko, Ivan P. Moskowitz

Dr. Linglin Xie should be included in the author byline instead of the Acknowledgments. She should be listed as the sixth author, and her affiliation is 1: Departments of Pediatrics, Pathology, and Human Genetics, The University of Chicago, Chicago, Illinois, United States of America. The contributions of this author are as follows: Investigation.

The correct citation is: Hoffmann AD, Yang XH, Burnicka-Turek O, Bosman JD, Ren X, Xie L, et al. (2014) *Foxf* Genes Integrate *Tbx5* and Hedgehog Pathways in the Second Heart Field for Cardiac Septation. PLoS Genet 10(10): e1004604. doi:10.1371/journal.pgen.1004604
