# Supplementary figures and images for: Foxf Genes Integrate Tbx5 and Hedgehog Pathways in the Second Heart Field for Cardiac Septation
Source: PLoS Genet. 2014 Oct 30;10(10):e1004604. doi: 10.1371/journal.pgen.1004604 (PMC4214600; doi:10.1371/journal.pgen.1004604)

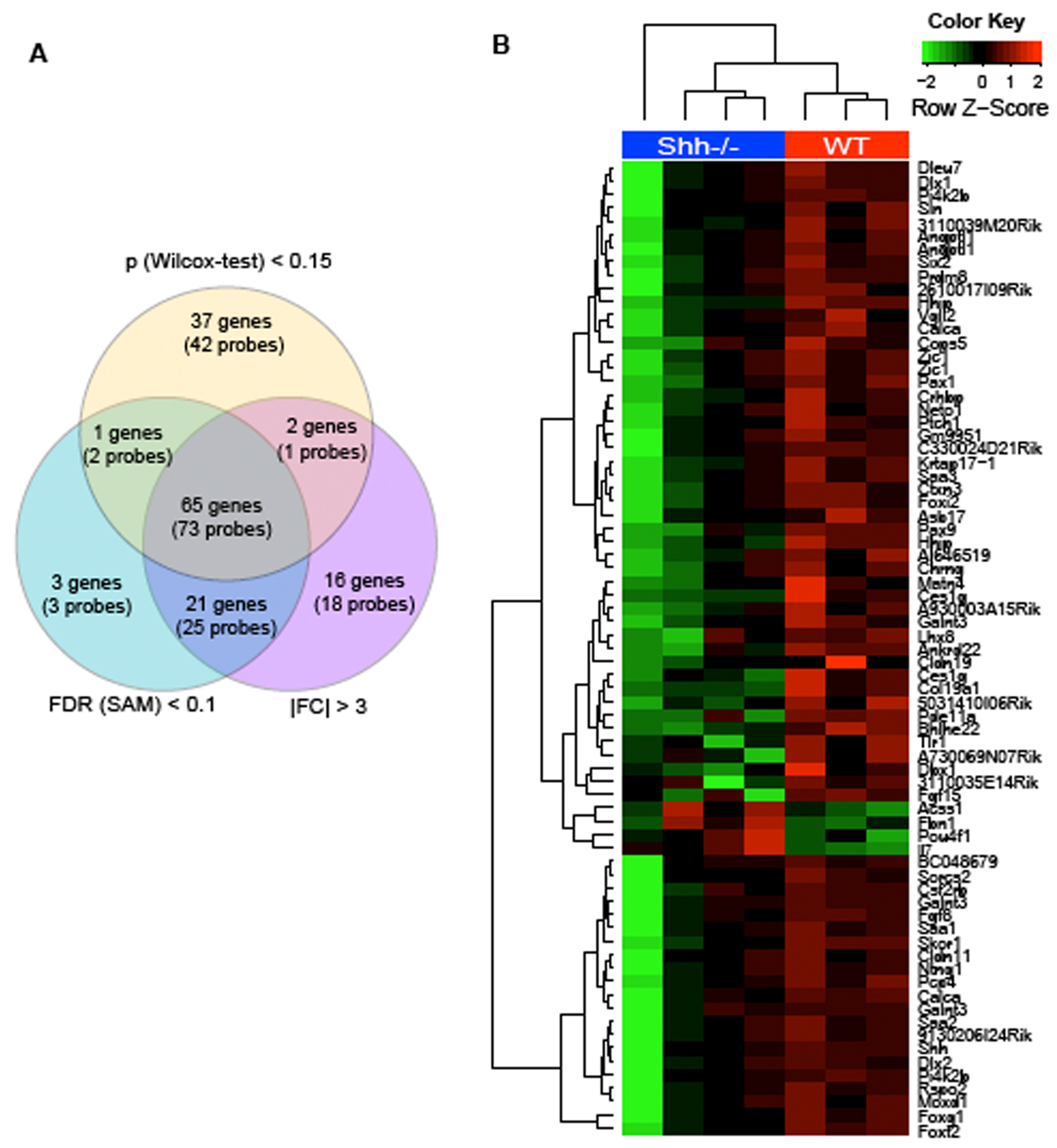

Supplement: Figure S1 — Optimized shh-dependent candidates for in vitro validation. Panel A) 65 genes generated from all three statistical tests on the same data are interrogated. Panel B) Hierarchical classification of samples based on the expression of these 65 genes splits Shh mutants from wild types. (TIF) [file pgen.1004604.s001.tif]

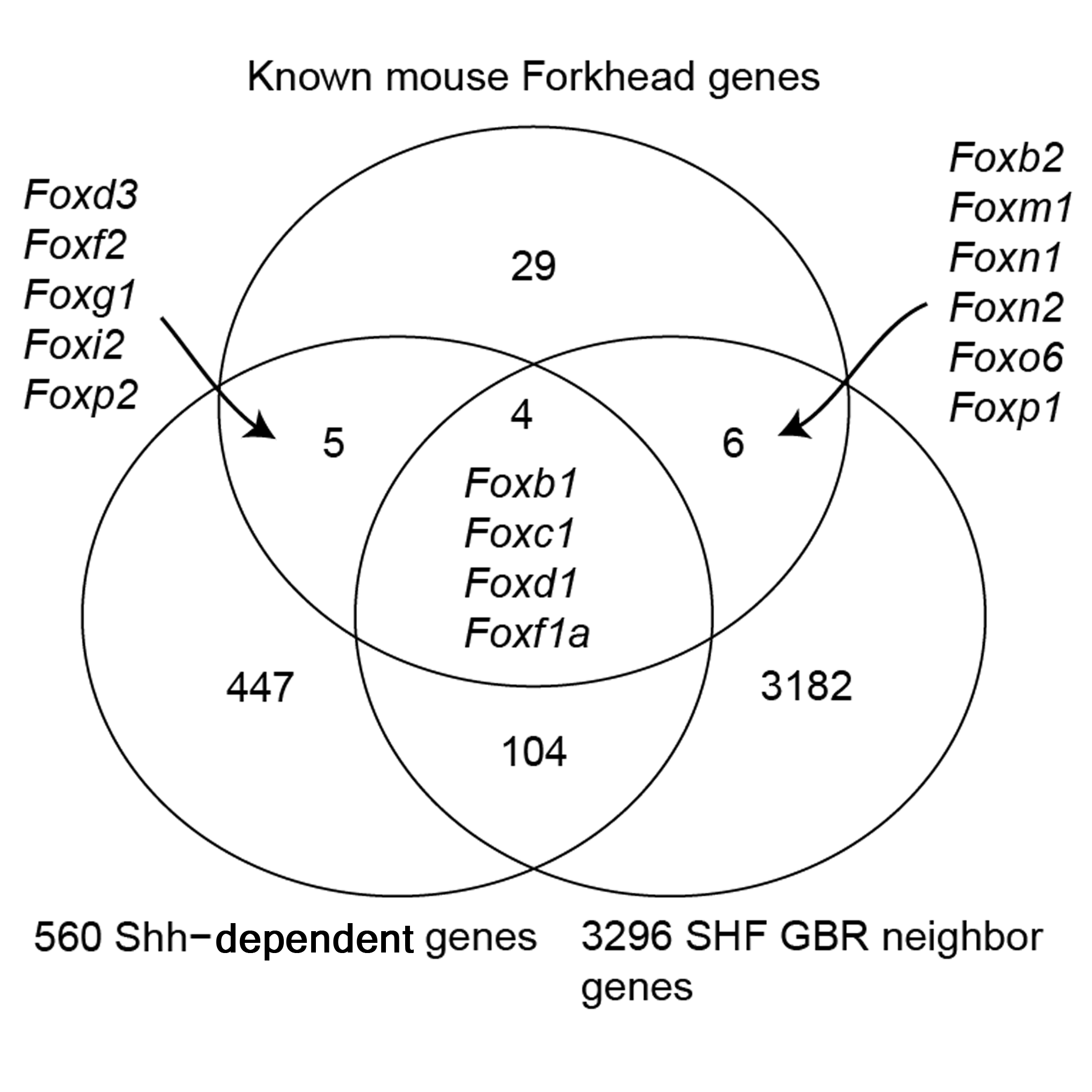

Supplement: Figure S2 — Known mouse Forkhead-box genes are enriched among the identified shh-dependent and Gli3T-bound genes. (TIF) [file pgen.1004604.s002.tif]

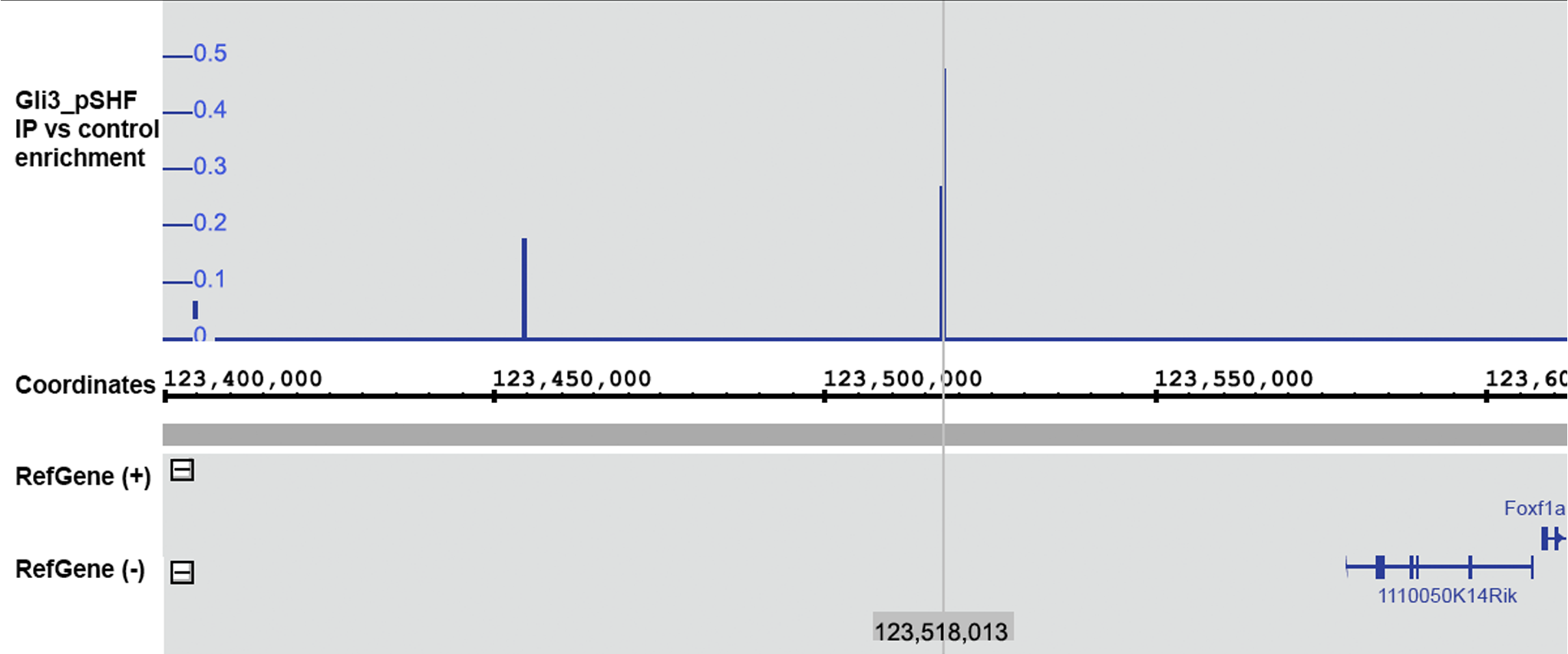

Supplement: Figure S3 — Browser views of the actual sequence mapping profiles between the identified Gli3-bound site and Foxf1a TSS. The two top panels show the estimated enriched peaks and the density measurements respectively. The two bottom panels are the genome (mm9) coordinates and the RefGenes. (TIF) [file pgen.1004604.s003.tif]

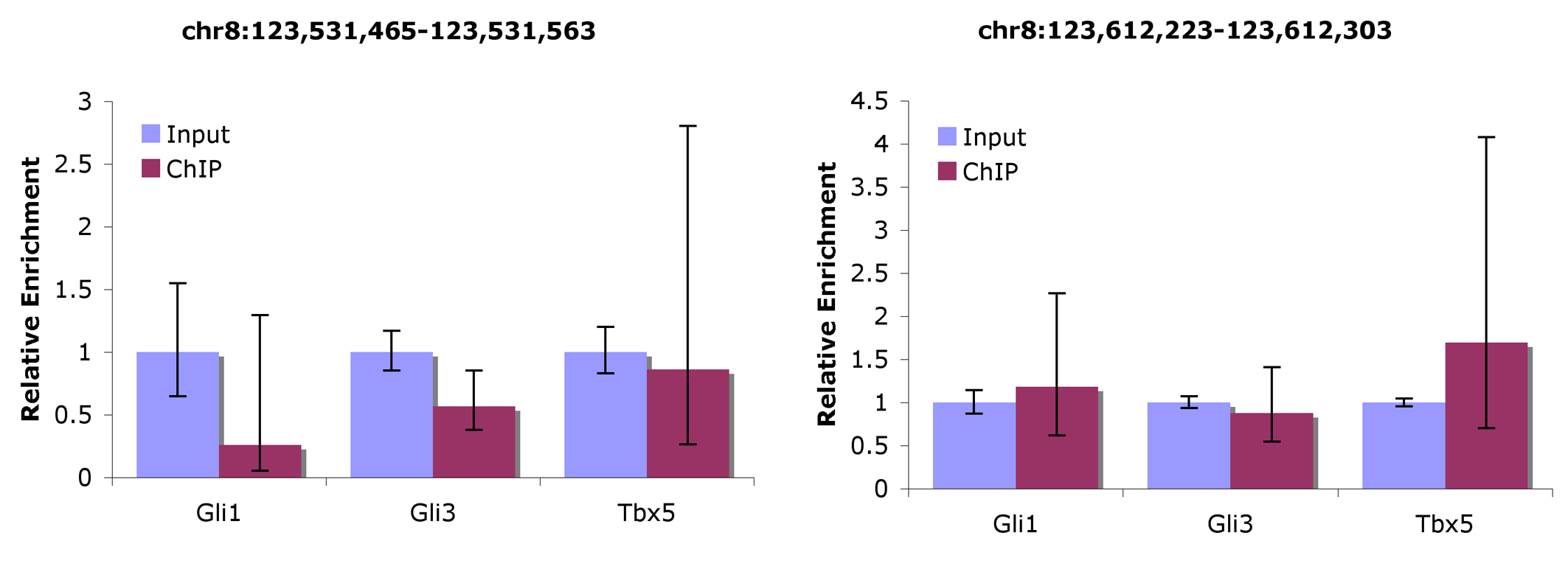

Supplement: Figure S4 — Genomic regions near the Foxf1a gene but without identified Gli or Tbx binding sites were tested in Gli1-Flag, Gli3-Flag, and Tbx5 ChIP samples as controls for specificity. Neither of these sites significantly amplified over input controls, suggesting that the Gli and Tbx5 ChIP was specific for the identified enhancer. (TIF) [file pgen.1004604.s004.tif]
